# Temporal trends in incidence, clinical characteristics and outcomes of pyogenic vertebral osteomyelitis in Southern Denmark from 2009 to 2022: a retrospective population-based cohort study

**DOI:** 10.1080/07853890.2026.2738249

**Published:** 2026-10-21

**Authors:** Susan Olaf Lindvig, Michala Kehrer, Inge Petersen, Søren Hess, Zahra Persson Theilgaard, Flemming Schønning Rosenvinge, Isik Somuncu Johansen

**Affiliations:** a Department of Infectious Diseases, Odense University Hospital, Odense, Denmark; b Department of Clinical Research, University of Southern Denmark, Odense, Denmark; c Department of Nuclear Medicine, Odense University Hospital, Odense, Denmark; d Department of Infectious Diseases, Copenhagen University Hospital, Rigshospitalet, Copenhagen, Denmark; e Department of Internal Medicine, Infectious Diseases Unit, Zealand University Hospital, Roskilde, Denmark; f Department of Clinical Microbiology, Odense University Hospital, Odense, Denmark; g Research Unit of Clinical Microbiology, University of Southern Denmark, Odense, Denmark

**Keywords:** Pyogenic vertebral osteomyelitis, cohort, incidence, epidemiology, temporal trends, mortality

## Abstract

**Objectives:**

Pyogenic vertebral osteomyelitis (PVO) is a severe infection with increasing incidence. We aimed to describe long-term population-based temporal trends in incidence, clinical presentation, diagnostic approaches, microbiology, and outcomes.

**Materials and methods:**

We conducted a retrospective population-based cohort study including 1,017 adults with multidisciplinary team–verified PVO diagnosed at a tertiary infectious diseases referral centre in the Region of Southern Denmark between 2009 and 2022. Incidence rates (IRs) per 100,000 person-years (PY) were calculated using regional population data and compared across three calendar periods (2009–2013, 2014–2018, 2019–2022). Temporal changes in clinical characteristics, microbiology, diagnostic strategies, and outcomes were assessed.

**Results:**

In total, 1,017 patients were included. The crude IR increased from 4.7 per 100,000 PY in 2009–2013 to 11.0 in 2019–2022, with the largest increase among patients aged ≥70 years. Mean age and comorbidity burden rose over time. Pain was common, fever was often absent, and neurological deficits occurred in 33.5% (*n* = 341). Postsurgical infection was the most frequent primary focus, and concomitant endocarditis occurred in 8.3% (*n* = 84). A microbiological diagnosis was established in 64.8% (*n* = 635) of cases, most commonly *Staphylococcus aureus*. Use of Fluorine-18 Fluorodeoxyglucose Positron Emission Tomography/Computed Tomography ([^18^F]FDG-PET/CT) increased over time. Median hospital stay remained 33 days. One-year mortality increased from 9.5% to 18.2%, while relapse declined, resulting in a stable composite risk of death or relapse (20.7%).

**Conclusions:**

The incidence of PVO increased markedly from 2009 to 2022, predominantly in older patients with comorbidities. Despite improved diagnostics, microbiologically-negative cases remain frequent. Mortality remained high and increased over time.

## KEY MESSAGES

The incidence of pyogenic vertebral osteomyelitis increased substantially over the 14-year study period, particularly in an ageing and increasingly comorbid population. Patients presented with signs of more advanced disease despite shorter symptom duration, while the persistently high proportion of microbiologically negative cases underscored ongoing diagnostic challenges. Sustained mortality and long-term pain highlight the considerable burden of PVO and the need for improved diagnostic pathways, risk stratification, and treatment strategies.

## Introduction

Pyogenic vertebral osteomyelitis (PVO) is a serious spinal infection associated with substantial morbidity, prolonged hospitalisation, and healthcare resource utilisation [1–5]. Although historically rare, the incidence rate (IR) has increased in recent decades, likely reflecting an ageing population, higher comorbidity, increased use of invasive procedures, and improved diagnostics [4–11]. In the Region of Southern Denmark, the incidence ranged from 2.2 to 5.8 per 100,000 population during 1995–2008, with a one-year mortality around 20% [1,6].

The clinical presentation of PVO is heterogeneous and often insidious. While some patients present acutely during bacteraemia, others report prolonged back pain with few systemic signs of infection. The distribution of these clinical phenotypes may be evolving, potentially driven by increasing bloodstream infections among older and more comorbid patients, increasing the risk of haematogenous seeding [12,13].

Despite advances in management, including evidence supporting shorter antibiotic courses in uncomplicated disease and increasing use of outpatient parenteral antimicrobial therapy, diagnostic delay remains common, and improvements in length of hospital stay (LOS) and long-term outcomes have been modest [14–18].

Population-based studies integrating clinical characteristics, microbiology, and outcomes remain limited, limiting insight into disease trends and prognosis. We therefore aimed to describe 14-year trends in PVO at a Danish university hospital, focusing on changes in incidence, patient characteristics, clinical presentation, microbiological aetiology, disease extent, recurrence and outcomes.

## Material and methods

### Study design and population

We conducted a retrospective cohort study using the Southern Denmark Cohort on Pyogenic Vertebral Infection (SPINE), including adults (≥18 years) diagnosed with PVO at the Department of Infectious Diseases, Odense University Hospital (OUH), Denmark, between 1 January 2009 and 31 December 2022.

OUH is the sole tertiary referral centre for infectious diseases in the Region of Southern Denmark, serving approximately 1.2 million residents. As PVO management is centralised at OUH, patients with suspected or confirmed PVO are referred for evaluation and treatment. The cohort therefore represents a population-based with near-complete regional case capture.

All PVO diagnoses were verified at the time of treatment through a multidisciplinary team (MDT) comprising infectious disease physicians, clinical microbiologists, spine surgeons, radiologists, and nuclear medicine specialists. The MDT meets weekly to confirm diagnoses and define treatment plans.

The study population included both native haematogenous and postoperative or implant-associated PVO. Exclusion criteria included fungal, mycobacterial, or brucella infections, a final diagnosis that could not be verified by imaging, and a diagnostic work-up exceeding five weeks. Patients were followed from the index admission for a minimum of 12 months.

### Data collection

Cases were initially identified using ICD-10 discharge codes (M465A; M463, M464) from the hospital registry. Only cases with an MDT-verified diagnosis were included in the final cohort. Electronic medical records were manually reviewed to extract data on demographics, clinical presentation, concomitant infections, recurrence and outcomes. Microbiological data were obtained from the Department of Clinical Microbiology and diagnostic imaging records were retrieved from the regional imaging archive.

Individual-level data sources were linked using the unique Danish Civil Registration Number (CRN). All data were entered into a REDCap^®^ database (Vanderbilt University, USA) hosted within the Open Patient Data Explorative Network (Region of Southern Denmark).

### Variables and definitions

Comorbidities were recorded as documented at the time of diagnosis and summarised using the Charlson Comorbidity Index (CCI) [19]. Microbiological aetiology was defined as pathogens detected by blood culture, culture of abscess material or tissue biopsy specimens, or obtained by 16S rDNA PCR from 14 days before to 30 days after first clinical contact. Reported microbiological findings were categorised as *Staphylococcus aureus* (including methicillin-sensitive and methicillin-resistant strains), coagulase-negative staphylococci (CoNS), *Cutibacterium acnes*, *Streptococcus* spp., other Gram-positive cocci, Enterobacterales, other Gram-negative bacilli, anaerobes, and polymicrobial. Microbiologically negative PVO was defined as the absence of pathogen identification despite microbiological investigation from any of the above-mentioned specimens.

Imaging findings from examinations performed between 14 days before and 30 days after first clinical contact were included to describe the extent of the infection and the presence of intraspinal or paravertebral abscesses. Neurological impairment was defined as any motor or sensory deficit documented at admission. Baseline laboratory parameters were recorded as the maximum value within ±7 days of admission, except for haemoglobin, which was recorded as the minimum value.

### Outcomes

The primary outcome was the temporal incidence of PVO across three predefined periods (2009–2014, 2015–2018, and 2019–2022) expressed per 100,000 (PY), using annual population data from Statistics Denmark [20].

Secondary outcomes included temporal trends in demographics, comorbidity burden, clinical presentation, microbiology and usage of diagnostic modalities. Clinical outcomes included LOS, one-year all-cause mortality and relapse. LOS was defined as the total number of days from index admission due to PVO related symptoms to hospital discharge. Relapses were defined as the occurrence of new or worsening clinical, microbiological, and/or radiological findings after completion of the initial treatment course, leading to re-initiation of antibiotic therapy following a treatment-free interval of at least 24 h and within one year of treatment completion.

### Statistical analysis

Summary statistics were reported as counts with percentages, or medians with interquartile ranges (IQR), as appropriate, for the overall cohort and stratified for three calendar periods (2009–2013, 2014–2018, and 2019–2022). Incidence rates (IR) were calculated per 100,000 (PY) using population data from Statistics Denmark [20] for all residents aged ≥18 years living in the Region of Southern Denmark on January 1 each of the relevant calendar years. For analysis covering multi-year periods, the mean population size across the years within each period was used as the denominator. Both crude and sex- and age-standardised (using year 2016 population weights) IR were estimated. Confidence intervals (CIs) for crude incidence rates (IRs) were estimated assuming a log-normal distribution. For standardised IRs, CIs were calculated using estimated standard errors derived from the stratum-specific standard errors, assuming binomial distributions within each stratum. The stratum-specific estimates were combined using the corresponding standardisation weights. Poisson regression models were applied to estimate incidence rate ratios (IRRs) and the average annual percentage change in incidence, with corresponding 95% CI. The risk of mortality within one-year was estimated using a Cox proportional regression model, adjusted for age, sex, and comorbidities. Furthermore, sensitivity analyses were performed to assess one-year mortality, incidence rates, and microbiological findings after excluding patients with a history of previous implants or back surgery. All statistical analyses were performed using Stata, version 18.0 (StataCorp. 2023, LLC., College Station, TX, USA).

### Ethics

The study was conducted under the general research approval granted by the Region of Southern Denmark (no. 18/18782) and the Danish Patient Safety Authority (no. 31-1521-296). In accordance with Danish legislation, registry-based observational studies do not require approval from the Regional Committees on Health Research Ethics, and written informed consent from participants is not required.

## Results

A total of 1,017 patients with PVO were included in the SPINE cohort after exclusion of 42 cases (Figure 1). The crude IR was 7.6 per 100,000 PY (95% CI 7.1–8.1). The crude IRs more than doubled between 2009–2013 and 2019–2022 (Figure 2a). The impact of direct sex- and age-standardisation of the IRs across the three time periods was virtually absent (Figure 2b). IRR, estimated from Poisson regression, showed the most pronounced increases among individuals aged 70–79 years (average annual increase in IR 6.6%, 95% CI 3.5–9.7) and aged ≥80 years (average annual increase 14.3%, 95% CI 10.1–18.6) (Figure 2c). The estimated annual increase in IR remained largely unchanged after adjustment for age and sex (IRR 1.07, 95% CI 1.05–1.08). Repeating the Poisson regression in a subsample of PVO patients without a history of implants or back surgeries had virtually no effect on the IRRs (results not shown).

**Figure 1.**
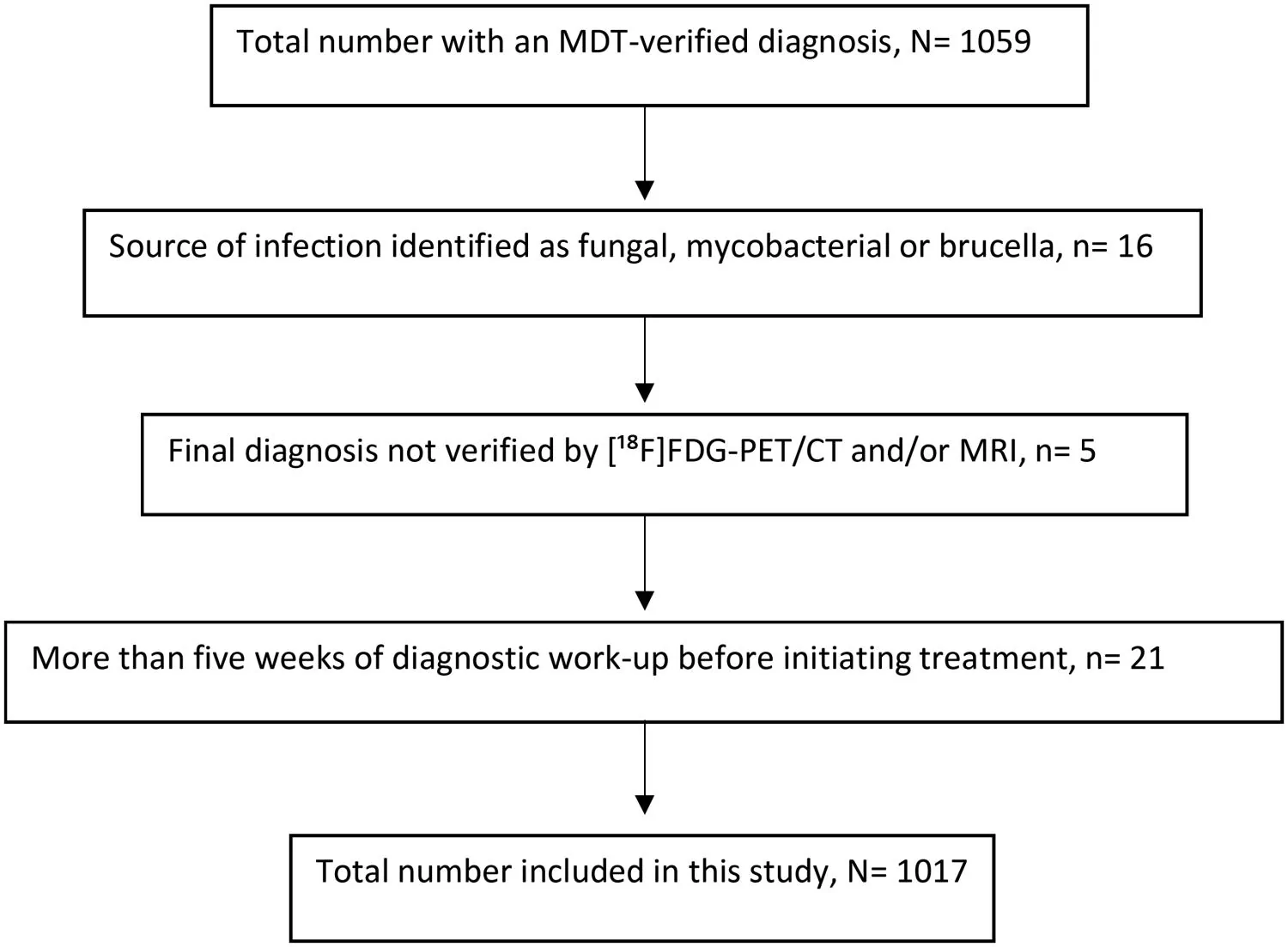
Flow diagram of patient inclusion in the SPINE cohort.

**Figure 2.**
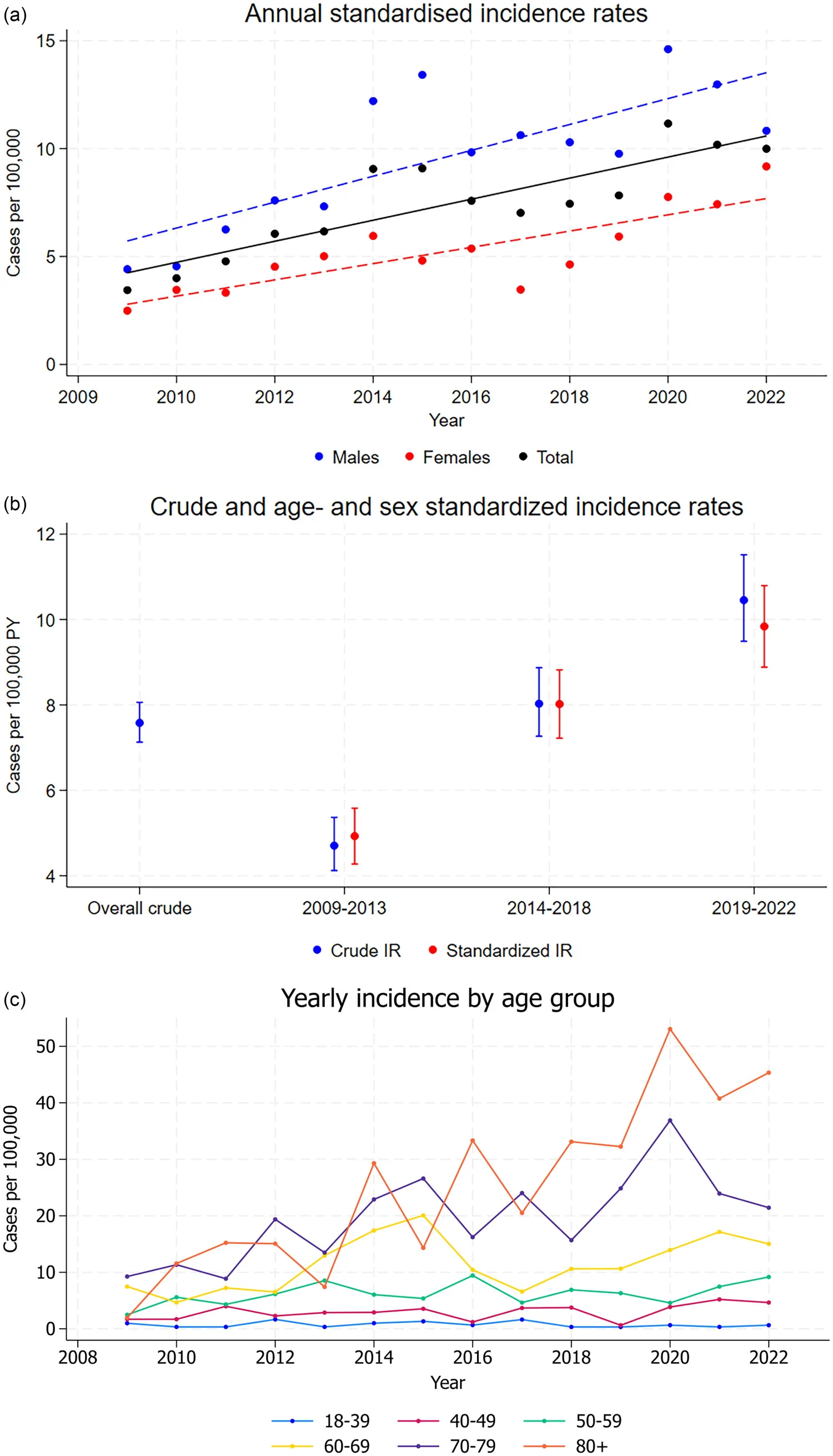
(a) Annual standardised incidence rates, (b) crude and age- and sex standardized incidence rates, and (c) yearly incidence by age group..

Baseline demographics and clinical characteristics are presented in Table 1. Males accounted for 64.5% (*n* = 656) of the cohort, and the median age at diagnosis was 70 (IQR 60–78) years. The median CCI score was 4 (IQR 3–6), with a rising comorbidity burden over time (Supplementary Material Table 1), and a slight rise in the proportion of patients with spinal implants was observed in the most recent period. Clinical presentation remained stable over time, with pain (91.1%, *n* = 926) and fever (45.7%, *n* = 465) as the most common symptoms. Neurological deficits were present in 33.5% (*n* = 341). Among patients admitted with back pain as the main symptom, 26.5% (*n* = 269) reported symptom duration exceeding three months prior to diagnosis. However, short symptom duration became more common over time, with the proportion of patients reporting symptoms for less than one week rising from 16.8% in the first period to 37% in the final period.

**Table 1.** Demographic and clinical characteristics at the time of diagnosis in the SPINE cohort, by time period.

	All (*N* = 1,017)	2009–2013 (*n* = 220)	2014–2018 (*n* = 386)	2019–2022 (*n* = 411)
Male	656 (64.5%)	133 (60.5%)	269 (69.7%)	254 (61.8%)
Age, median (IQR)	70 (60–78)	66 (55–74.5)	69 (60–78)	74 (63–80)
Age groups				
18–39	32 (3.1%)	11 (5.0%)	15 (3.9%)	6 (1.5%)
40–49	69 (6.8%)	22 (10.0%)	25 (6.5%)	22 (5.4%)
50–59	147 (14.5%)	44 (20.0%)	55 (14.2%)	48 (11.7%)
60–69	245 (24.1%)	59 (26.8%)	100 (25.9%)	86 (20.9%)
70–80	310 (30.5%)	57 (25.9%)	116 (30.1%)	137 (33.3%)
80+	214 (21.0%)	27 (12.3%)	75 (19.4%)	112 (27.3%)
Charlson co-morbidity score (IQR)	4 (3–6)	3 (2–5)	4 (3–6)	5 (3–7)
Previous spinal implants	53 (5.2%)	8 (3.6%)	19 (4.9%)	26 (6.3%)
Symptoms at admission				
Fever	465 (45.7%)	78 (35.5%)	178 (46.1%)	209 (50.9%)
Pain	926 (91.1%)	196 (89.1%)	349 (90.4%)	381 (92.7%)
Neurological symptoms	341 (33.5%)	72 (32.7%)	107 (27.7%)	162 (39.4%)
Weight loss	193 (19.0%)	45 (20.5%)	85 (22.0%)	63 (15.3%)
Back pain as main symptom and cause of admission				
No	236 (23.2%)	44 (20.0%)	94 (24.4%)	98 (23.8%)
Acute pain (<3 months)	503 (49.5%)	108 (49.1%)	185 (47.9%)	210 (51.1%)
Pain lasting >3 months	269 (26.5%)	65 (29.5%)	105 (27.2%)	99 (24.1%)
Neurological symptoms				
Weakness in limbs	140 (13.8%)	36 (16.4%)	39 (10.1%)	65 (15.8%)
Sensory loss or numbness in limbs	81 (8.0%)	19 (8.6%)	29 (7.5%)	33 (8.0%)
Decreased ability to walk	117 (11.5%)	22 (10.0%)	37 (9.6%)	58 (14.1%)
Urine or fecal incontinence	153 (15.0%)	24 (10.9%)	41 (10.6%)	88 (21.4%)
Duration of symptoms				
Less than 1 week	288 (28.3%)	37 (16.8%)	99 (25.6%)	152 (37.0%)
1–4 weeks	245 (24.1%)	61 (27.7%)	101 (26.2%)	83 (20.2%)
1–3 months	161 (15.8%)	39 (17.7%)	64 (16.6%)	58 (14.1%)
More than 3 months	172 (16.9%)	45 (20.5%)	67 (17.4%)	60 (14.6%)
Primary focus of infection				
Skin/soft tissue infection (incl. intravenous drug use)	76 (7.5%)	16 (7.3%)	33 (8.5%)	27 (6.6%)
Odontogenic infection	29 (2.9%)	8 (3.6%)	11 (2.8%)	10 (2.4%)
Post-surgical	90 (8.8%)	18 (8.2%)	30 (7.8%)	42 (10.2%)
Infected intravascular devices	35 (3.4%)	6 (2.7%)	13 (3.4%)	16 (3.9%)
Urinary tract infection	65 (6.4%)	8 (3.6%)	23 (6.0%)	34 (8.3%)
Concomitant infection				
Endocarditis	84 (8.3%)	20 (9.1%)	20 (5.2%)	44 (10.7%)
Meningitis	15 (1.5%)	4 (1.8%)	6 (1.6%)	5 (1.2%)
Incidental finding	94 (9.2%)	13 (5.9%)	37 (9.6%)	44 (10.7%)
LOS days (IQR)	33 (21–47)	33 (15.5-47)	33 (22–49)	32 (21–44)

Abbreviations: IQR, interquartile range; LOS, length of hospital stay.

A suspected primary infectious focus was identified in a minority of patients, with postsurgical infections accounting for 8.8% (*n* = 90) of cases and an increasing proportion over time. Concomitant endocarditis was present in 8.3% (*n* = 84) of cases, whereas meningitis remained rare. 9.2% (*n* = 94) of cases were diagnosed incidentally during evaluation for other conditions. Median LOS remained stable across the study period at 33 days (IQR 21–47) (Table 1).

Imaging findings by magnetic resonance imaging (MRI) and Fluorine-18 Fluorodeoxyglucose Positron Emission Tomography/Computed Tomography ([^18^F]FDG-PET/CT) are presented in Table 2. MRI was the initial diagnostic modality in 66% (*n* = 671) of cases, although its use as first-line imaging declined over time. MRI was performed in 89.6% (*n* = 911) of cases, with findings consistent with PVO observed in 97.8% of the examinations. Intraspinal and paravertebral abscesses were identified in 27.1% and 28.5%, respectively. Phlegmonous changes were common and increasingly reported over time, while psoas abscesses were identified in 19.8% of MRI examinations.

**Table 2.** Imaging modalities and findings at time of diagnosis in the SPINE cohort, by time period.

Initial diagnostic imaging method*	All (*N* = 1,017)	2009–2013 (*n* = 220)	2014–2018 (*n* = 386)	2019–2022 (*n* = 411)
MRI	671 (66.0%)	171 (77.7%)	247 (64.0%)	253 (61.6%)
[¹⁸F]FDG-PET/CT	346 (34.0%)	49 (22.3%)	139 (36.0%)	158 (38.4%)
MRI				
Imaging performed	911 (89.6%)	200 (90.9%)	347 (89.9%)	364 (88.6%)
Indicating PVO	891 (97.8%)	198 (99.0%)	340 (98.0%)	353 (97.0%)
Intraspinal abscess	247 (27.1%)	57 (28.5%)	89 (25.6%)	101 (27.7%)
Paravertebral abscess	260 (28.5%)	50 (25.0%)	102 (29.4%)	108 (29.7%)
Intraspinal phlegmon	268 (29.4%)	20 (10.0%)	92 (26.5%)	156 (42.9%)
Paravertebral phlegmon	288 (31.6%)	21 (10.5%)	105 (30.3%)	162 (44.5%)
Psoas abscess	180 (19.8%)	33 (16.5%)	77 (22.2%)	70 (19.2%)
[¹⁸F]FDG-PET/CT				
Imaging performed	791 (77.8%)	111 (50.5%)	334 (86.5%)	346 (84.2%)
Indicating PVO	784 (99.1%)	108 (97.3%)	331 (99.1%)	345 (99.7%)
Intraspinal abscess	49 (6.2%)	5 (4.5%)	17 (5.1%)	27 (7.8%)
Paravertebral abscess	85 (10.7%)	6 (5.4%)	48 (14.4%)	31 (9.0%)
Intraspinal phlegmon	60 (7.6%)	0 (0.0%)	7 (2.1%)	53 (15.3%)
Paravertebral phlegmon	146 (18.5%)	5 (4.5%)	41 (12.3%)	100 (28.9%)
Psoas abscess	141 (17.8%)	23 (20.7%)	66 (19.8%)	52 (15.0%)
Location of spinal lesions				
Cervical	96 (9.4%)	21 (9.5%)	35 (9.1%)	40 (9.7%)
Thoracic	180 (17.7%)	36 (16.4%)	62 (16.1%)	82 (20.0%)
Lumbar	559 (55.0%)	134 (60.9%)	206 (53.4%)	219 (53.3%)
Sacral	1 (0.1%)	0 (0.0%)	0 (0.0%)	1 (0.2%)
Multiple locations	181 (17.8%)	29 (13.2%)	83 (21.5%)	69 (16.8%)

* First diagnostic modality to raise suspicion of PVO. Abbreviations: MRI, Magnetic Resonance Imaging; [¹⁸F]FDG-PET/CT, Fluorine-18 Fluorodeoxyglucose Positron Emission Tomography/Computed Tomography; PVO, pyogenic vertebral osteomyelitis.

[^18^F]FDG-PET/CT was performed in 77.8% (*n* = 791) of cases, and 99.1% of the examinations showed findings consistent with PVO. Abscess detection rates were lower than with MRI, with intraspinal abscesses identified in 6.2%, paravertebral abscesses in 10.7%, and psoas abscesses in 17.8% of scans.

Lumbar involvement was most frequent (55%), followed by thoracic and cervical localisation. Laboratory results are presented in Table 3. Data completeness improved over time due to increasing digitalisation of laboratory records, from approximately one third of patients in 2009–2013 to more than 90% in later periods. Anaemia was common, affecting 82.7% (*n* = 836) of patients, and white blood cell and neutrophil counts were frequently elevated. C-reactive protein levels were markedly increased, with a median value of 188 mg/L. Erythrocyte sedimentation rate was elevated in nearly all tested patients, whereas alkaline phosphatase generally remained within the normal range.

**Table 3.** Laboratory findings at time of diagnosis in the SPINE cohort, by time period.

	Proportion tested (across all periods)	All (*N* = 1017)	2009–2013 (*n* = 220)	2014–2018 (*n* = 386)	2019–2022 (*n* = 411)
Hemoglobin (mmol/L)	82.2%	6.3 (5.5–7.2) (*n* = 836)	6.6 (5.8–7.4) (*n* = 68)	6.3 (5.4–7.2) (*n* = 371)	6.2 (5.5–7.1) (*n* = 397)
Proportion tested			30.9%	96.1%	96.6%
Anemia (<7.5 mmol/L)		691/836 (82.7%)	53/68 (77.9%)	296/371 (79.8%)	342/397 (86.1%)
White blood cell count (10^9^ mol/L)	82.4%	13.1 (10.2–17.4) (*n* = 838)	13.9 (10.2–18.9) (*n* = 68)	12.7 (10.1–17.2) (*n* = 372)	13.4 (10.2–17.4) (*n* = 398)
Proportion tested			30.9%	96.4%	96.8%
Elevated WBC count (>9.0 × 10^9^ mol/L)		711/838 (84.8%)	58/68 (85.3%)	312/372 (83.9%)	341/398 (85.7%)
Neutrophils (10^9^ mol/L)	77.7%	9.8 (7.2–14.1) (*n* = 790)	10.6 (7.0–14.9) (*n* = 61)	9.4 (6.7–13.6) (*n* = 340)	10.1 (7.5–14.4) (*n* = 389)
Proportion tested			27.7%	88.1%	94.6%
Elevated neutrophils (>6.0 × 10^9^ mol/L)		656/790 (83.0%)	50/61 (82.0%)	274/340 (80.6%)	332/389 (85.3%)
Thrombocytes (10^9^ mol/L)	78.5%	337.0 (248.0–450.0) (*n* = 798)	342.5 (270.0–475.0) (*n* = 60)	346.0 (260.0–446.5) (*n* = 352)	332.0 (230.0–454.0) (*n* = 386)
Proportion tested			27.3%	91.2%	93.9%
Elevated thrombocytes (>400 × 10^9^ mol/L)		282/798 (35.3%)	25/60 (41.7%)	124/352 (35.2%)	133/386 (34.5%)
C-reactive protein (mg/L)	82.3%	188.0 (99.0–291.0) (*n* = 837)	139.5 (53.5–273.0) (*n* = 68)	201.5 (99.5–293.0) (*n* = 372)	185.0 (110.0–289.0) (*n* = 397)
Proportion tested			30.9%	96.4%	96.6%
Low C-reactive protein (≤10 mg/L)		20/837 (2.4%)	6/68 (8.8%)	7/372 (1.9%)	7/397 (1.8%)
Elevated C-reactive protein (10–100 mg/L)		190/837 (22.7%)	19/68 (27.9%)	86/372 (23.1%)	85/397 (21.4%)
Elevated C-reactive protein (>100 mg/L)		627/837 (74.9%)	43/68 (63.2%)	279/372 (75.0%)	305/397 (76.8%)
Erythrocyte sedimentation rate (mm/h)	15.3%	82.0 (53.5–96.0) (*n* = 156)	67.0 (39.0–83.0) (*n* = 22)	83.0 (57.0–95.0) (*n* = 79)	85.0 (64.0–97.0) (*n* = 55)
Proportion tested			10.0%	20.5%	13.4%
Elevated sedimentation rate (>20 mm/h)		146/156 (93.6%)	20/22 (90.9%)	75/79 (94.9%)	51/55 (92.7%)
Alkaline phosphate (U/L)	75.9%	29.0 (18.0–54.0) (*n* = 772)	29.0 (18.0–60.0) (*n* = 58)	29.0 (17.0–57.0) (*n* = 349)	30.0 (18.0–50.0) (*n* = 365)
Proportion tested			26.4%	90.4%	88.8%
Elevated alkaline phosphate (>105 U/L)		68/772 (8.8%)	3/58 (5.2%)	39/349 (11.2%)	26/365 (7.1%)

Data are presented as median (interquartile range) and *n* (%). Percentages are calculated based on the number of patients with available data. “Proportion tested” indicates the proportion of patients with available measurements in each time period.

Microbiological findings are presented in Table 4. A bacterial pathogen was identified by culture or PCR from blood, pus, or tissue biopsy in 64.8% (*n* = 635) of patients. *S. aureus* was the most common pathogen (41.2%), followed by *Streptococcus* spp. (18.5%) and Enterobacterales (11.7%). Exclusion of patients with implants or previous back surgery did not materially change the proportion of patients with a positive microbiological diagnosis (64.8% vs. 64.0%) or the overall distribution of bacterial pathogens. (Data not shown). No microbiological diagnosis based on blood, pus or tissue biopsy samples was established in 35.8% (*n* = 382) of patients.

**Table 4.** Microbiological findings^a^ at time of diagnosis in the SPINE cohort, by time period.

	All (*n* = 1017)	2009–2013 (*n* = 220)	2014–2018 (*n* = 386)	2019–2022 (*n* = 411)
All				
Tested	980 (96.4%)	205 (93.2%)	372 (96.4%)	403 (98.1%)
Positive test	635 (64.8%)	116 (56.6%)	246 (66.1%)	273 (67.7%)
Blood culture				
Tested	979 (96.3%)	204 (92.7%)	372 (96.4%)	403 (98.1%)
Positive culture (%)	622 (63.5%)	110 (53.9%)	241 (64.8%)	271 (67.2%)
Bacteria				
*Staphylococcus aureus* (Including methicillin-sensitive and methicillin-resistant strains)	256 (41.2%)	48 (43.6%)	89 (36.9%)	119 (43.9%)
Coagulase-negative staphylococci (CoNS)	56 (9.0%)	11 (10.0%)	26 (10.8%)	19 (7.0%)
*Streptococcus* spp.	115 (18.5%)	18 (16.4%)	46 (19.1%)	51 (18.8%)
Other Gram-positive cocci	46 (7.4%)	10 (9.1%)	17 (7.1%)	19 (7.0%)
*Cutibacterium acnes*	5 (0.8%)	0 (0.0%)	5 (2.1%)	0 (0.0%)
Enterobacterales	73 (11.7%)	11 (10.0%)	25 (10.4%)	37 (13.7%)
Other gram-negative bacilli	9 (1.4%)	2 (1.8%)	4 (1.7%)	3 (1.1%)
Anaerobes	4 (0.6%)	1 (0.9%)	2 (0.8%)	1 (0.4%)
Polymicrobial	58 (9.3%)	9 (8.2%)	27 (11.2%)	22 (8.1%)
Pus cultures or tissue biopsy cultures				
Tested	114 (11.2%)	33 (15.0%)	38 (9.8%)	43 (10.5%)
Positive test	71 (62.3%)	19 (57.6%)	25 (65.8%)	27 (62.8%)
Bacteria				
*Staphylococcus aureus* (Including methicillin-sensitive and methicillin-resistant strains)	44 (62.0%)	10 (52.6%)	19 (76.0%)	15 (55.6%)
Coagulase-negative staphylococci (CoNS)	2 (2.8%)	1 (5.3%)	0 (0.0%)	1 (3.7%)
*Streptococcus* spp.	6 (8.5%)	0 (0.0%)	1 (4.0%)	5 (18.5%)
Other Gram-positive cocci	4 (5.6%)	2 (10.5%)	0 (0.0%)	2 (7.4%)
*Cutibacterium acnes*	1 (1.4%)	1 (5.3%)	0 (0.0%)	0 (0.0%)
Enterobacterales	6 (8.5%)	3 (15.8%)	2 (8.0%)	1 (3.7%)
Other Gram-negative bacilli	1 (1.4%)	1 (5.3%)	0 (0.0%)	0 (0.0%)
Anaerobes	2 (2.8%)	0 (0.0%)	1 (4.0%)	1 (3.7%)
Polymicrobial	5 (7.0%)	1 (5.3%)	2 (8.0%)	2 (7.4%)

^a^ Cultures of blood, abscess material or tissue biopsy specimens, and 16S rDNA PCR and sequencing.

Outcomes are summarised in Table 5. At last follow-up, persistent pain requiring analgesics was reported in 23.9% (*n* = 243) of patients, increasing from 19.5% to 26.5% over time. Residual neurological symptoms, including weakness, sensory loss, or numbness, were uncommon. One-year all-cause mortality was 13.6% and increased from 9.5% to 18.2% across the study periods. The one-year relapse rate was 8.1%, with the highest rate observed in 2014–2018 and a marked decline in the most recent period. However, the overall composite risk of one-year mortality or relapse was 20.7% throughout the study period. Hazard Ratios (HR) from Cox regression adjusted for age, sex, and comorbidities demonstrated a statistically non-significant lower risk of one-year mortality risk during 2014–2018 compared with the preceding and subsequent periods in the full sample. However, in the sample excluding patients with implants or back surgery, the HRs increased over the three time periods, although the differences remained statistically non-significant.

**Table 5.** Clinical outcomes in the SPINE cohort, by time period.

	All (*N* = 1,017)	2009–2013 (*n* = 220)	2014–2018 (*n* = 386)	2019–2022 (*n* = 411)
Pain necessitating analgesic use	243 (23.9%)	43 (19.5%)	91 (23.6%)	109 (26.5%)
Weakness in limbs	26 (2.6%)	10 (4.5%)	11 (2.8%)	5 (1.2%)
Sensory loss or numbness in limbs	24 (2.4%)	7 (3.2%)	8 (2.1%)	9 (2.2%)
One-year mortality (*n* (%))	138 (13.6%)	21 (9.5%)	42 (10.9%)	75 (18.2%)
One-year mortality* (HR (95% CI))	NA	1 (Ref)	0.77 (0.46–1.31)	1.08 (0.66–1.77)
One-year mortality,* (HR (95% CI)) in reduced sample**	NA	1 (Ref)	1.17 (0.56–2.41)	1.91 (0.96–3.78
One-year relapse	82 (8.1%)	18 (8.2%)	52 (13.5%)	12 (2.9%)
One-year mortality or relapse	211 (20.7%)	39 (17.7%)	89 (23.1%)	83 (20.2%)

* Hazard ratio adjusted for age, sex and grouped Charlson comorbidity score (score = 0, 1–2, 3–5, 6–15).

** Patients without implants or previous back surgery.

## Discussion

In this population-based cohort study, we observed a marked and sustained increase in the incidence of PVO over the 14-year study period. The incidence increased by an average of 8.0% per year (95% CI 6.3–9.7), coinciding with an ageing population and an increasing burden of comorbidity. Over time, patients more frequently presented with neurological involvement and other markers of disease severity. Although relapse rates declined substantially in recent years, one-year mortality remained high.

The increasing incidence is consistent with reports from several European countries and Danish studies and likely reflects demographic shifts towards older populations with a higher prevalence of chronic comorbid conditions [1,2,5,6,8–11]. In England, hospital admissions for PVO rose 44% between 2012 and 2021, with the largest increase among individuals aged 75–89 years [21]. Similarly, German registry data indicate that incidence reached 11 cases per 100,000 population in 2021, corresponding to a 104% increase since 2005 [9]. In line with these trends, our cohort demonstrated a substantial and persistent increase in comorbidity burden, reflected by rising CCI scores. Diabetes and other chronic conditions were prevalent and have previously been associated with poorer outcomes, including increased mortality [3,6,15,17].

Pain remained a near-universal presenting symptom, consistent with prior studies [3,4,17,21,22]. Neurological involvement increased slightly over time, which could be suggestive of presentation at a more advanced stage. Fever was reported in only half of patients, broadly consistent with previous reports [6,21,23]. These patterns highlight the ongoing challenges of timely diagnosis of PVO in routine clinical practice.

Symptom duration before diagnosis declined over the study period. This finding may be explained by several factors, including increased clinical awareness, improvements in diagnostic methods, and earlier diagnostic work-up. However, it could also reflect an ageing and increasingly medically complex population, which may be more likely to present with more acute or severe symptoms, leading to a faster presentation rather than a true biological change in disease progression [21,22].

A primary infection focus was identified in a subset of patients, most commonly post-surgical, skin/soft tissue, or urinary tract infections. Post-surgical cases likely represent direct inoculation rather than haematogenous seeding. Concomitant infective endocarditis was observed in 8% of patients, comparable to prior reports [7,23].

Median LOS remained stable at 33 days (IQR 21–47), which is longer than reported in cohorts from England and Germany [5,11]. This may reflect a higher burden of comorbidity, prolonged use of intravenous antibiotic therapy [8], and the continued reliance on in-hospital multidisciplinary management despite increasing availability of outpatient parenteral antimicrobial therapy in Denmark.

MRI remained the cornerstone of diagnosis throughout the study period. More frequent reporting of intraspinal and paravertebral inflammatory changes over time may reflect advances in imaging quality and more systematic imaging assessment but could also indicate increasing disease severity. Use of [^18^F]FDG-PET/CT increased substantially and is now commonly used to complement MRI and aiding in assessment of disease extent [24]. The lumbar spine was most frequently affected, followed by the thoracic and cervical regions, consistent with previous studies [8,25].

Laboratory findings consistently demonstrated pronounced systemic inflammation, with a high proportion of patients presenting with markedly elevated CRP levels (>100 mg/L), this may indicate a trend towards more advanced disease at presentation.

Despite advances in microbiological diagnostics, a considerable proportion of cases remain microbiologically unverified. Sampling from the infection site also varies across studies, reflecting differences in diagnostic practice [6,17,21,26,27]. This underlines the persistent challenge of microbiological confirmation and the consequent reliance on empirical, non-tailored antimicrobial therapy.

Persistent pain requiring analgesics was common and increased from 19.5% to 26.5% over the study period, consistent with previous reports highlighting chronic pain as a frequent sequela of PVO [10,28]. This increase may reflect improved recognition of chronic pain, longer survival with residual symptoms, or more advanced disease at diagnosis.

One-year mortality was comparable to Danish and international cohorts during 2009–2018 [10,21,23], but increased to 18% in the most recent period, while relapse rates declined over time. These divergent trends may reflect more consistent treatment strategies alongside demographic shifts towards an older and more comorbid population. Changes in case ascertainment or healthcare system factors may also have contributed, underscoring the persistent severity of PVO.

Key strengths of this study include the large, unselected population-based design, long-term follow-up, and systematic multidisciplinary verification of diagnoses, enhancing completeness and internal validity.

Limitations include the retrospective design, which precludes causal inference. Changes in coding practices, access to imaging, laboratory data and population demographics may have influenced case ascertainment. Symptom onset and neurological findings relied on medical records and may be subject to information bias. Despite extensive validation, some misclassification cannot be excluded.

## Conclusion

Over the 14-year study period, the incidence of PVO increased substantially in parallel with an ageing and increasingly comorbid population. Patients appeared to present clinical features consistent with more advanced disease, despite a shorter reported symptom duration. The persistently high proportion of microbiologically negative cases highlights ongoing diagnostic challenges, while sustained mortality and long-term pain underscore the considerable burden of this clinical complex condition. These findings highlight the need for improved diagnostic pathways, better risk stratification, and optimised treatment strategies to improve outcomes of patients with PVO.

## Supplementary Material

AoM_Supplementary table 1.docx

## Data Availability

The data supporting the findings in this article are available within the article and its supplementary table.
