# Supplementary material for: Temporal trends in incidence, clinical characteristics and outcomes of pyogenic vertebral osteomyelitis in Southern Denmark from 2009 to 2022: a retrospective population-based cohort study
Source: Ann Med. 2026 Oct 21;58(1):2738249. doi: 10.1080/07853890.2026.2738249 (PMC13689801; doi:10.1080/07853890.2026.2738249)
Supplement: Supplementary file 1 [file IANN_A_2738249_SM2288.docx]

Supplementary table 1: Co-morbidities at time of diagnosis in the SPINE-cohort, by time period.

|  | **All** | **2009-2014** | **2015-2018** | **2019-2022** |
| --- | --- | --- | --- | --- |
| **Co-morbidities** |  |  |  |  |
| Myocardial infarct | 155 (15.2%) | 29 (13.2%) | 54 (14.0%) | 72 (17.5%) |
| Congestive heart failure | 84 (8.3%) | 12 (5.5%) | 33 (8.5%) | 39 (9.5%) |
| Peripheral vascular disease | 433 (42.6%) | 53 (24.1%) | 157 (40.7%) | 223 (54.3%) |
| Cerebrovascular disease | 106 (10.4%) | 18 (8.2%) | 34 (8.8%) | 54 (13.1%) |
| Dementia | 33 (3.2%) | 3 (1.4%) | 12 (3.1%) | 18 (4.4%) |
| Chronic pulmonary disease | 154 (15.1%) | 21 (9.5%) | 67 (17.4%) | 66 (16.1%) |
| Connective tissue disease | 96 (9.4%) | 18 (8.2%) | 45 (11.7%) | 33 (8.0%) |
| Ulcer disease | 42 (4.1%) | 9 (4.1%) | 24 (6.2%) | 9 (2.2%) |
| Mild liver disease | 24 (2.4%) | 5 (2.3%) | 10 (2.6%) | 9 (2.2%) |
| Diabetes (without complications) | 172 (16.9%) | 26 (11.8%) | 67 (17.4%) | 79 (19.2%) |
| Diabetes with end organ damage | 31 (3.0%) | 2 (0.9%) | 11 (2.8%) | 18 (4.4%) |
| Hemiplegia | 17 (1.7%) | 2 (0.9%) | 7 (1.8%) | 8 (1.9%) |
| Moderate or severe renal disease | 112 (11.0%) | 25 (11.4%) | 45 (11.7%) | 42 (10.2%) |
| Solid tumor (non metastatic) | 75 (7.4%) | 11 (5.0%) | 27 (7.0%) | 37 (9.0%) |
| Leukemia | 6 (0.6%) | 2 (0.9%) | 1 (0.3%) | 3 (0.7%) |
| Lymphoma, Multiple myeloma | 15 (1.5%) | 2 (0.9%) | 6 (1.6%) | 7 (1.7%) |
| Moderate or severe liver disease | 45 (4.4%) | 11 (5.0%) | 15 (3.9%) | 19 (4.6%) |
| Metastatic solid tumor | 39 (3.8%) | 2 (0.9%) | 12 (3.1%) | 25 (6.1%) |
| AIDS | 0 (0.0%) | 0 (0.0%) | 0 (0.0%) | 0 (0.0%) |

*Data are presented as n (%). Percentages are calculated based on available data. Abbreviations: AIDS, acquired immunodeficiency syndrome.*
